# 5,8-Dimeth­oxy-2-phenyl-1,4-dihydro­quinoline-3-carbonitrile

**DOI:** 10.1107/S1600536810031685

**Published:** 2010-08-18

**Authors:** Souheila Ladraa, Abdelmalek Bouraiou, Sofiane Bouacida, Thierry Roisnel, Ali Belfaitah

**Affiliations:** a Laboratoire des Produits Naturels d’Origine Végétale et de Synthèse Organique, PHYSYNOR, Université Mentouri-Constantine, 25000 Constantine, Algeria; b Unité de Recherche de Chimie de l’Environnement, et Moléculaire Structurale, CHEMS, Université Mentouri-Constantine, 25000 Algeria; c Centre de Difractométrie X, UMR 6226 CNRS Unité Sciences Chimiques de Rennes, Université de Rennes I, 263 Avenue du Général Leclerc, 35042 Rennes, France

## Abstract

The crystal structure of the title mol­ecule, C_18_H_16_N_2_O_2_, can be described as two types of crossed layers parallel to the (110) and (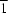
10) planes. An intra­molecular N—H⋯O hydrogen bond occurs.

## Related literature

For our previous work on the preparation of quinoline derivatives see: Benzerka *et al.* (2008 ▶); Ladraa *et al.* (2009 ▶, 2010 ▶); Moussaoui *et al.* (2002 ▶); Menasra *et al.* (2005 ▶); Belfaitah *et al.* (2006 ▶); Bouraiou *et al.* (2006 ▶, 2007 ▶, 2008 ▶). For more details of quinoline reduction, see: Dauphinee & Forrest (1978 ▶); Srikrishna *et al.* (1996 ▶); Vierhapper & Eliel (1975 ▶); Lim *et al.* (1995 ▶).
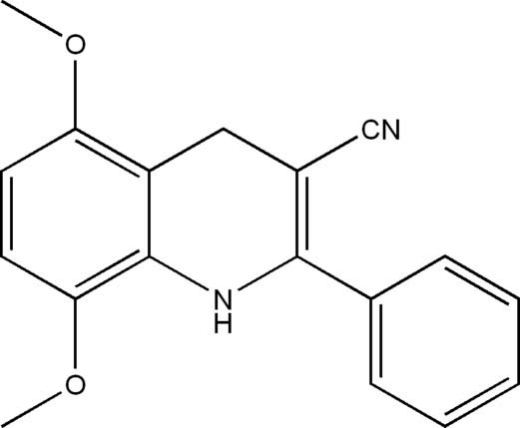


## Experimental

### 

#### Crystal data

C_18_H_16_N_2_O_2_
*M* *_r_* = 292.33
Monoclinic, 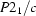

*a* = 3.9952 (3) Å
*b* = 20.4544 (15) Å
*c* = 17.7313 (13) Å
β = 95.976 (5)°
*V* = 1441.12 (18) Å^3^
*Z* = 4
Mo *K*α radiation
μ = 0.09 mm^−1^
*T* = 150 K
0.27 × 0.07 × 0.05 mm

#### Data collection

Bruker APEXII diffractometer
Absorption correction: multi-scan (*SADABS*: Sheldrick, 2002 ▶) *T*_min_ = 0.702, *T*_max_ = 0.996
12491 measured reflections
3292 independent reflections
1975 reflections with *I* > 2σ(*I*)
*R*_int_ = 0.051

#### Refinement

*R*[*F* ^2^ > 2σ(*F* ^2^)] = 0.049
*wR*(*F* ^2^) = 0.133
*S* = 1.03
3292 reflections
201 parameters
H-atom parameters constrained
Δρ_max_ = 0.17 e Å^−3^
Δρ_min_ = −0.29 e Å^−3^

### 

Data collection: *APEX2* (Bruker, 2001 ▶); cell refinement: *SAINT* (Bruker, 2001 ▶); data reduction: *SAINT*; program(s) used to solve structure: *SIR2002* (Burla *et al.*, 2003 ▶); program(s) used to refine structure: *SHELXL97* (Sheldrick, 2008 ▶); molecular graphics: *ORTEP-3 for Windows* (Farrugia, 1997 ▶) and *DIAMOND* (Brandenburg & Berndt, 2001 ▶); software used to prepare material for publication: *WinGX* (Farrugia, 1999 ▶).

## Supplementary Material

Crystal structure: contains datablocks global, I. DOI: 10.1107/S1600536810031685/hg2695sup1.cif

Structure factors: contains datablocks I. DOI: 10.1107/S1600536810031685/hg2695Isup2.hkl

Additional supplementary materials:  crystallographic information; 3D view; checkCIF report

## Figures and Tables

**Table 1:** Hydrogen-bond geometry (Å, °)

*D*—H⋯*A*	*D*—H	H⋯*A*	*D*⋯*A*	*D*—H⋯*A*
N1—H1⋯O1	0.88	2.29	2.649 (2)	104

**Fig. 1.**
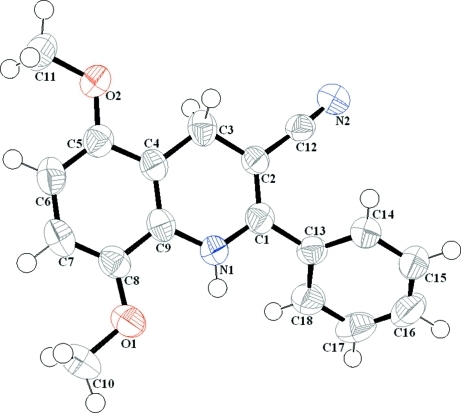
(Farrugia, 1997) the structure of the title compound with the atomic labelling scheme. Displacement are drawn at the 50% probability level.

**Fig. 2.**
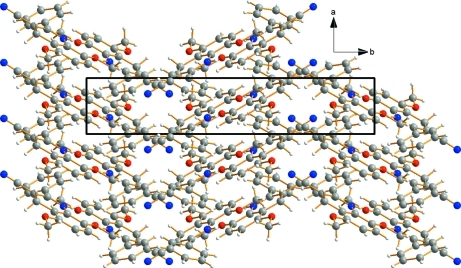
(Brandenburg & Berndt, 2001) A diagram of the layered crystal packing of (I) viewed down the c axis.

## supplementary crystallographic information

### Comment

In quinoline and its derivatives it is usually the pyridine ring which is reduced first. Sodium in liquid ammonia converted quinoline to 1,2-dihydroquinoline (Dauphinee *et al.*, 1978). 1,2,3,4-tetrahydroquinoline was obtained by catalytic hydrogenation and by reduction with borane and sodium cyanoborohydride (Srikrishna *et al.*, 1996), 5,6,7,8-tetrhydroquinoline by catalytic hydrogenation over platinum oxide or 5% palladium or rhodium on carbon in triflouroacetic acid (Vierhapper *et al.*, 1975). Vigorous hydrogenation gave *cis* and *trans*-decahydroquinoline. The reducing proprieties of hydrazine are due to its thermal decomposition to hydrogen and nitrogen. The heating of hydrazine with aromatic hydrocarbons at 160–280°C effected complete hydrogenation of the aromatic ring. On the other hand, zinc is used to a limited extent for reductions of double bonds conjugated with strongly polar groups and partial reduction of some aromatics. The majority of reductions with zinc are carried out in acids: hydrochloric, sulfuric, formic and especially acetic. In previous works, we were interested in the design and synthesis of new molecules that contain a quinolyl moiety (Benzerka *et al.*, 2008; Ladraa *et al.*, 2009, 2010, Moussaoui *et al.*, 2002; Menasra *et al.*, 2005; Belfaitah *et al.*, 2006 and Bouraiou *et al.*,2006, 2007, 2008). In this paper, we report the structure determination of new compound that result from an unwanted reduction of the pyridine ring of 3-cyano-5,8-dimethoxy-2-phenylquinoline. Our attempt to create a tetrazine ring linked quinolyl moiety, using hydrazine in the presence of Cu(NO_3_)_2_.3H_2_O-Zn, was failed and led to 1,4-dihydro-5,8-dimethoxy-2-phenylquinoline-3-carbonitrile (I).

The molecular geometry and the atom-numbering scheme of (I) are shown in Fig. 1. The asymmetric unit of title compound contains a 1,4-dihydroquinolyl unit bearing a phenyl ring at position C-2, nitril group at C-3 and two methoxy at C-5 and C-8. The two rings of 1,4-dihydroquinolyl moiety are fused in an axial fashion and form a dihedral angle of 0.17 (5)°. The phenyl ring form also with quinolyl plane a dihedral angle of 45.38 (6)°. The crystal packing can be described by two types of crossed layers which 1,4-dihydroquinolyl ring is parallel to (110) and (-110)planes respectively (Fig. 2). The crystal packing is stabilized by intramolecular hydrogen bond (N—H···O) and Van der Waals interactions, resulting in the formation of a three-dimensional network and reinforcing a cohesion of structure. Hydrogen-bonding parameters are listed in table 1.

### Experimental

Compound (I) was obtained by modification of reported procedure (Lim *et al.*, 1995). Refluxing a mixture of 1 eq. of 3-cyano-5,8-dimethoxy-2-phenylquinoline, 2 eq. of zinc and 1 eq. of Cu(NO_2_)_2_. 3H_2_O in the presence of 4 eq. of hydrazine monohydrate for 3 days lead to the corresponding 1,4-dihydro-5,8-dimethoxy-2-phenylquinoline-3-carbonitrile I. The product was purified by column chromatography. Single crystals suitable for X-ray diffraction analysis were obtained by dissolving the corresponding compound in CH_2_Cl_2_/Petroleum ether mixture and letting the solution for slow evaporation at room temperature.

### Refinement

All non-H atoms were refined with anisotropic atomic displacement parameters. All H atoms were localized on Fourier maps but introduced in calculated positions and treated as riding on their parent C atom.

### Crystal data

**Table:**

C_18_H_16_N_2_O_2_	*F*(000) = 616
*M**_r_* = 292.33	*D*_x_ = 1.347 Mg m^−^^3^
Monoclinic, *P*2_1_/*c*	Mo *K*α radiation, λ = 0.71073 Å
Hall symbol: -P 2ybc	Cell parameters from 2693 reflections
*a* = 3.9952 (3) Å	θ = 2.3–25.3°
*b* = 20.4544 (15) Å	µ = 0.09 mm^−^^1^
*c* = 17.7313 (13) Å	*T* = 150 K
β = 95.976 (5)°	Stick, colourless
*V* = 1441.12 (18) Å^3^	0.27 × 0.07 × 0.05 mm
*Z* = 4	

### Data collection

**Table:**

Bruker APEXII diffractometer	1975 reflections with *I* > 2σ(*I*)
graphite	*R*_int_ = 0.051
CCD rotation images, thin slices scans	θ_max_ = 27.6°, θ_min_ = 2.5°
Absorption correction: multi-scan (*SADABS*: Sheldrick, 2002)	*h* = −3→5
*T*_min_ = 0.702, *T*_max_ = 0.996	*k* = −26→24
12491 measured reflections	*l* = −22→22
3292 independent reflections	

### Refinement

**Table:**

Refinement on *F*^2^	Primary atom site location: structure-invariant direct methods
Least-squares matrix: full	Secondary atom site location: difference Fourier map
*R*[*F*^2^ > 2σ(*F*^2^)] = 0.049	Hydrogen site location: inferred from neighbouring sites
*wR*(*F*^2^) = 0.133	H-atom parameters constrained
*S* = 1.03	*w* = 1/[σ^2^(*F*_o_^2^) + (0.0462*P*)^2^ + 0.6371*P*] where *P* = (*F*_o_^2^ + 2*F*_c_^2^)/3
3292 reflections	(Δ/σ)_max_ < 0.001
201 parameters	Δρ_max_ = 0.17 e Å^−^^3^
0 restraints	Δρ_min_ = −0.29 e Å^−^^3^

### Special details

**Table:**

Geometry. All e.s.d.'s (except the e.s.d. in the dihedral angle between two l.s. planes) are estimated using the full covariance matrix. The cell e.s.d.'s are taken into account individually in the estimation of e.s.d.'s in distances, angles and torsion angles; correlations between e.s.d.'s in cell parameters are only used when they are defined by crystal symmetry. An approximate (isotropic) treatment of cell e.s.d.'s is used for estimating e.s.d.'s involving l.s. planes.
Refinement. Refinement of *F*^2^ against ALL reflections. The weighted *R*-factor *wR* and goodness of fit *S* are based on *F*^2^, conventional *R*-factors *R* are based on *F*, with *F* set to zero for negative *F*^2^. The threshold expression of *F*^2^ > σ(*F*^2^) is used only for calculating *R*-factors(gt) *etc*. and is not relevant to the choice of reflections for refinement. *R*-factors based on *F*^2^ are statistically about twice as large as those based on *F*, and *R*- factors based on ALL data will be even larger.

### Fractional atomic coordinates and isotropic or equivalent isotropic displacement parameters (Å^2^)

**Table:**

	*x*	*y*	*z*	*U*_iso_*/*U*_eq_	
C1	0.1215 (5)	0.13620 (9)	0.66975 (11)	0.0267 (5)	
C2	0.0694 (5)	0.18185 (9)	0.72449 (11)	0.0252 (4)	
C3	0.2040 (5)	0.17402 (10)	0.80517 (11)	0.0302 (5)	
H3A	0.0148	0.1763	0.8370	0.036*	
H3B	0.3573	0.2110	0.8198	0.036*	
C4	0.3890 (5)	0.11164 (9)	0.82153 (11)	0.0258 (4)	
C5	0.5299 (5)	0.09642 (10)	0.89555 (11)	0.0280 (5)	
C6	0.7054 (5)	0.03884 (10)	0.91013 (12)	0.0329 (5)	
H6	0.7993	0.0289	0.9603	0.040*	
C7	0.7448 (5)	−0.00496 (10)	0.85068 (12)	0.0329 (5)	
H7	0.8668	−0.0444	0.8609	0.040*	
C8	0.6090 (5)	0.00849 (9)	0.77773 (11)	0.0282 (5)	
C10	0.8195 (6)	−0.09023 (10)	0.72813 (14)	0.0402 (6)	
H10A	1.0483	−0.0807	0.7509	0.060*	
H10B	0.8296	−0.1130	0.6798	0.060*	
H10C	0.7053	−0.1180	0.7626	0.060*	
C11	0.6472 (6)	0.13355 (11)	1.02389 (11)	0.0377 (5)	
H11A	0.5699	0.0926	1.0450	0.056*	
H11B	0.5963	0.1701	1.0566	0.056*	
H11C	0.8906	0.1313	1.0211	0.056*	
C12	−0.1237 (5)	0.23988 (10)	0.70655 (11)	0.0273 (5)	
C13	0.0034 (5)	0.14369 (9)	0.58792 (11)	0.0268 (4)	
C14	0.0519 (5)	0.20237 (10)	0.55075 (11)	0.0319 (5)	
H14	0.1607	0.2378	0.5779	0.038*	
C15	−0.0572 (6)	0.20951 (11)	0.47439 (12)	0.0383 (6)	
H15	−0.0232	0.2498	0.4496	0.046*	
C16	−0.2153 (6)	0.15839 (12)	0.43412 (13)	0.0419 (6)	
H16	−0.2928	0.1637	0.3820	0.050*	
C17	−0.2606 (6)	0.09925 (12)	0.46989 (12)	0.0409 (6)	
H17	−0.3670	0.0639	0.4421	0.049*	
C18	−0.1503 (5)	0.09145 (11)	0.54663 (12)	0.0347 (5)	
H18	−0.1794	0.0507	0.5709	0.042*	
C9	0.4271 (5)	0.06718 (9)	0.76313 (11)	0.0265 (4)	
N1	0.2916 (4)	0.07985 (8)	0.68900 (9)	0.0294 (4)	
H1	0.3166	0.0506	0.6536	0.035*	
N2	−0.2790 (5)	0.28731 (9)	0.69710 (10)	0.0370 (5)	
O1	0.6360 (4)	−0.02995 (7)	0.71508 (8)	0.0351 (4)	
O2	0.4784 (4)	0.14325 (7)	0.94924 (8)	0.0337 (4)	

### Atomic displacement parameters (Å^2^)

**Table:**

	*U*^11^	*U*^22^	*U*^33^	*U*^12^	*U*^13^	*U*^23^
C1	0.0277 (11)	0.0253 (11)	0.0271 (11)	−0.0058 (8)	0.0032 (8)	0.0022 (8)
C2	0.0292 (11)	0.0213 (10)	0.0250 (10)	−0.0039 (8)	0.0028 (8)	0.0011 (8)
C3	0.0297 (11)	0.0285 (11)	0.0324 (11)	−0.0049 (9)	0.0028 (9)	0.0070 (9)
C4	0.0272 (11)	0.0220 (10)	0.0287 (11)	−0.0034 (8)	0.0052 (8)	0.0045 (8)
C5	0.0298 (11)	0.0280 (11)	0.0262 (11)	−0.0047 (8)	0.0029 (8)	0.0023 (8)
C6	0.0361 (13)	0.0304 (12)	0.0318 (12)	−0.0007 (9)	0.0007 (9)	0.0089 (9)
C7	0.0331 (12)	0.0252 (11)	0.0407 (13)	0.0033 (9)	0.0047 (9)	0.0083 (9)
C8	0.0294 (11)	0.0226 (10)	0.0335 (11)	−0.0027 (8)	0.0070 (8)	0.0023 (8)
C10	0.0401 (14)	0.0264 (12)	0.0548 (15)	0.0061 (10)	0.0088 (11)	−0.0030 (10)
C11	0.0463 (14)	0.0409 (13)	0.0245 (11)	−0.0055 (10)	−0.0022 (9)	0.0027 (9)
C12	0.0341 (12)	0.0240 (11)	0.0240 (10)	−0.0047 (9)	0.0044 (8)	−0.0027 (8)
C13	0.0271 (11)	0.0275 (11)	0.0258 (11)	0.0017 (8)	0.0033 (8)	−0.0019 (8)
C14	0.0389 (13)	0.0288 (11)	0.0287 (11)	0.0025 (9)	0.0061 (9)	0.0010 (9)
C15	0.0514 (15)	0.0366 (13)	0.0279 (12)	0.0090 (11)	0.0092 (10)	0.0048 (9)
C16	0.0462 (15)	0.0560 (16)	0.0233 (11)	0.0130 (12)	0.0022 (10)	−0.0021 (10)
C17	0.0403 (14)	0.0482 (15)	0.0332 (12)	0.0000 (11)	−0.0009 (10)	−0.0140 (11)
C18	0.0380 (13)	0.0311 (12)	0.0347 (12)	−0.0034 (9)	0.0030 (9)	−0.0031 (9)
C9	0.0262 (11)	0.0225 (10)	0.0309 (11)	−0.0038 (8)	0.0043 (8)	0.0050 (8)
N1	0.0406 (11)	0.0229 (9)	0.0246 (9)	0.0000 (7)	0.0025 (7)	0.0010 (7)
N2	0.0475 (12)	0.0287 (10)	0.0348 (10)	0.0044 (9)	0.0045 (8)	−0.0014 (8)
O1	0.0422 (9)	0.0258 (8)	0.0378 (9)	0.0044 (6)	0.0071 (7)	−0.0003 (6)
O2	0.0436 (9)	0.0320 (8)	0.0244 (8)	0.0003 (6)	−0.0019 (6)	0.0000 (6)

### Geometric parameters (Å, °)

**Table:**

C1—N1	1.363 (3)	C10—H10B	0.9800
C1—C2	1.378 (3)	C10—H10C	0.9800
C1—C13	1.486 (3)	C11—O2	1.435 (2)
C2—C12	1.433 (3)	C11—H11A	0.9800
C2—C3	1.484 (3)	C11—H11B	0.9800
C3—C4	1.488 (3)	C11—H11C	0.9800
C3—H3A	0.9900	C12—N2	1.154 (3)
C3—H3B	0.9900	C13—C14	1.393 (3)
C4—C9	1.398 (3)	C13—C18	1.400 (3)
C4—C5	1.408 (3)	C14—C15	1.386 (3)
C5—O2	1.381 (2)	C14—H14	0.9500
C5—C6	1.381 (3)	C15—C16	1.381 (3)
C6—C7	1.405 (3)	C15—H15	0.9500
C6—H6	0.9500	C16—C17	1.386 (3)
C7—C8	1.377 (3)	C16—H16	0.9500
C7—H7	0.9500	C17—C18	1.395 (3)
C8—O1	1.374 (2)	C17—H17	0.9500
C8—C9	1.413 (3)	C18—H18	0.9500
C10—O1	1.441 (2)	C9—N1	1.393 (2)
C10—H10A	0.9800	N1—H1	0.8800
			
N1—C1—C2	120.29 (17)	O2—C11—H11A	109.5
N1—C1—C13	115.53 (17)	O2—C11—H11B	109.5
C2—C1—C13	124.18 (18)	H11A—C11—H11B	109.5
C1—C2—C12	121.48 (17)	O2—C11—H11C	109.5
C1—C2—C3	122.67 (18)	H11A—C11—H11C	109.5
C12—C2—C3	115.85 (17)	H11B—C11—H11C	109.5
C2—C3—C4	113.79 (17)	N2—C12—C2	175.5 (2)
C2—C3—H3A	108.8	C14—C13—C18	119.03 (19)
C4—C3—H3A	108.8	C14—C13—C1	120.34 (17)
C2—C3—H3B	108.8	C18—C13—C1	120.61 (18)
C4—C3—H3B	108.8	C15—C14—C13	120.5 (2)
H3A—C3—H3B	107.7	C15—C14—H14	119.7
C9—C4—C5	118.87 (18)	C13—C14—H14	119.7
C9—C4—C3	120.23 (17)	C16—C15—C14	120.4 (2)
C5—C4—C3	120.90 (18)	C16—C15—H15	119.8
O2—C5—C6	124.87 (17)	C14—C15—H15	119.8
O2—C5—C4	114.55 (17)	C15—C16—C17	119.9 (2)
C6—C5—C4	120.58 (19)	C15—C16—H16	120.1
C5—C6—C7	119.86 (19)	C17—C16—H16	120.1
C5—C6—H6	120.1	C16—C17—C18	120.2 (2)
C7—C6—H6	120.1	C16—C17—H17	119.9
C8—C7—C6	120.88 (19)	C18—C17—H17	119.9
C8—C7—H7	119.6	C17—C18—C13	120.0 (2)
C6—C7—H7	119.6	C17—C18—H18	120.0
O1—C8—C7	126.04 (18)	C13—C18—H18	120.0
O1—C8—C9	114.84 (17)	N1—C9—C4	121.09 (17)
C7—C8—C9	119.11 (19)	N1—C9—C8	118.22 (18)
O1—C10—H10A	109.5	C4—C9—C8	120.68 (18)
O1—C10—H10B	109.5	C1—N1—C9	121.88 (17)
H10A—C10—H10B	109.5	C1—N1—H1	119.1
O1—C10—H10C	109.5	C9—N1—H1	119.1
H10A—C10—H10C	109.5	C8—O1—C10	116.18 (16)
H10B—C10—H10C	109.5	C5—O2—C11	116.77 (16)
			
N1—C1—C2—C12	−176.67 (19)	C13—C14—C15—C16	−0.1 (3)
C13—C1—C2—C12	3.5 (3)	C14—C15—C16—C17	−1.0 (3)
N1—C1—C2—C3	2.6 (3)	C15—C16—C17—C18	0.7 (3)
C13—C1—C2—C3	−177.25 (19)	C16—C17—C18—C13	0.7 (3)
C1—C2—C3—C4	−2.0 (3)	C14—C13—C18—C17	−1.8 (3)
C12—C2—C3—C4	177.34 (17)	C1—C13—C18—C17	179.85 (19)
C2—C3—C4—C9	0.9 (3)	C5—C4—C9—N1	179.80 (18)
C2—C3—C4—C5	−179.41 (18)	C3—C4—C9—N1	−0.5 (3)
C9—C4—C5—O2	−179.88 (16)	C5—C4—C9—C8	−1.2 (3)
C3—C4—C5—O2	0.4 (3)	C3—C4—C9—C8	178.53 (19)
C9—C4—C5—C6	0.6 (3)	O1—C8—C9—N1	0.9 (3)
C3—C4—C5—C6	−179.07 (19)	C7—C8—C9—N1	−179.98 (18)
O2—C5—C6—C7	−179.32 (19)	O1—C8—C9—C4	−178.15 (17)
C4—C5—C6—C7	0.1 (3)	C7—C8—C9—C4	1.0 (3)
C5—C6—C7—C8	−0.3 (3)	C2—C1—N1—C9	−2.1 (3)
C6—C7—C8—O1	178.81 (19)	C13—C1—N1—C9	177.77 (17)
C6—C7—C8—C9	−0.2 (3)	C4—C9—N1—C1	1.1 (3)
N1—C1—C13—C14	−133.8 (2)	C8—C9—N1—C1	−178.01 (18)
C2—C1—C13—C14	46.0 (3)	C7—C8—O1—C10	1.1 (3)
N1—C1—C13—C18	44.5 (3)	C9—C8—O1—C10	−179.86 (18)
C2—C1—C13—C18	−135.6 (2)	C6—C5—O2—C11	6.0 (3)
C18—C13—C14—C15	1.5 (3)	C4—C5—O2—C11	−173.46 (18)
C1—C13—C14—C15	179.86 (19)		

### Hydrogen-bond geometry (Å, °)

**Table:**

*D*—H···*A*	*D*—H	H···*A*	*D*···*A*	*D*—H···*A*
N1—H1···O1	0.88	2.29	2.649 (2)	104
